# Emerging roles and regulation of MiT/TFE transcriptional factors

**DOI:** 10.1186/s12964-018-0242-1

**Published:** 2018-06-15

**Authors:** Min Yang, En Liu, Li Tang, Yuanyuan Lei, Xuemei Sun, Jiaxi Hu, Hui Dong, Shi-Ming Yang, Mingfa Gao, Bo Tang

**Affiliations:** 1 Department of Gastroenterology, Xinqiao Hospital, Third Military Medical University (Army Medical University), Chongqing, 400037 China; 2 Department of Medicine, University of California San Diego, San Diego, CA 92093 USA; 3 Department of Hepatobiliary Surgery, Xinqiao Hospital, Third Military Medical University (Army Medical University), Chongqing, 40037 China

**Keywords:** TFEB, TFE3, MiT/TFE family, Lysosome, Autophagy, Cancer

## Abstract

The MiT/TFE transcription factors play a pivotal role in the regulation of autophagy and lysosomal biogenesis. The subcellular localization and activity of MiT/TFE proteins are primarily regulated through phosphorylation. And the phosphorylated protein is retained in the cytoplasm and subsequently translocates to the nucleus upon dephosphorylation, where it stimulates the expression of hundreds of genes, leading to lysosomal biogenesis and autophagy induction. The transcription factor-mediated lysosome-to-nucleus signaling can be directly controlled by several signaling molecules involved in the mTORC1, PKC, and AKT pathways. MiT/TFE family members have attracted much attention owing to their intracellular clearance of pathogenic factors in numerous diseases. Recently, multiple studies have also revealed the MiT/TFE proteins as master regulators of cellular metabolic reprogramming, converging on autophagic and lysosomal function and playing a critical role in cancer, suggesting that novel therapeutic strategies could be based on the modulation of MiT/TFE family member activity. Here, we present an overview of the latest research on MiT/TFE transcriptional factors and their potential mechanisms in cancer.

## Background

The Microphthalmia family of bHLH-LZ transcription factors (MiT/TFE) is composed of four members. Previous studies reveal that the MiT/TFE transcriptional factors play important roles in the regulation of cellular processes. In particular, recent evidence suggests that the MiT/TFE family members might function as critical factors in cancer. In this review, we focus on the novel mechanism involved in the regulation of the activation of MiT/TFE transcriptional factors and their role in lysosomal homeostasis and autophagy induction. We also describe the participation of the aberrant activation of MiT/TFE members in cancer and discuss the potential therapeutic strategy in cancer.

## MiT/TFE family of transcription factors

The Microphthalmia family of bHLH-LZ transcription factors (MiT/TFE) is composed of four members: MITF, TFEB, TFE3 and TFEC [1, 2]. All members of the MiTF/TFE family share a similar structure that includes three critically important regions. The basic motif, which is required for DNA binding, and highly similar Helix-loop-helix (HLH) and leucine-zipper (Zip) regions are important for their dimerization; however, outside of these regions, these proteins are quite different [3]. Besides, TFEB, TFE3 and MITF also contain a conserved activation domain that is important for their transcriptional activation [1], while the activation domain is missing in TFEC, which is the most divergent member of the family and appears to inhibit, rather than activate, transcription [4].

Homodimerization and heterodimerization within the members of the MiTF/TFE family is critical for binding to DNA and the transcriptional activation of target genes [5]. MiT/TFE members bind the palindromic CACGTG E-box, a motif also recognized by other bHLH-Zip transcription factors, such as MYC, MAX and MAD proteins [6]. Unlike other bHLH-Zip transcription factors, MiT/TFE proteins also specifically bind the asymmetric TCATGTG M-box response elements present in the promoter region of their downstream target genes [7]. However, MiT/TFE proteins do not heterodimerize with other bHLH-Zip-containing proteins directly, such as MYC and USF [5, 6]. A previous study demonstrated that a conserved three-residue shift within the Zip domain of the MiT/TFE members generates an unusual out-of-register leucine zipper that allows for specific heterodimerization among MiT/TFE members, while preventing binding to other bHLH-Zip transcription factors [5]. However, the functional relevance of MiT/TFE homodimers compared to heterodimers remains unknown.

All four MiT/TFE members are conserved in vertebrates [8]. The MITF gene is predominantly expressed in the retinal pigment epithelium (RPE), macrophages, osteoclasts, mast cells, melanocytes and natural killer cells [2], while TFEC expression is restricted to cells of myeloid origin [9]. In contrast, TFE3 and TFEB show a more ubiquitous pattern of expression and have been detected in multiple cell types [10]. A large body of evidence suggests that the expression of MITF isoforms (this expression is different in the amino-terminal regions) is due to alternative splicing and posttranslational modifications [11]. Many of these isoforms show a tissue-dependent manner due to the usage of alternative promoters. For example, MITF-M is mainly expressed in melanoblasts and melanocytes, MITF-D is preferentially expressed in monocyte lineages [12], and MITF-A and MITF-H can be detected in several cells [3]. Analyzing their molecular structures, we conclude that TFEB and TFEC contain multiple alternative first exons with restricted and differential tissue distributions, whereas the TFE3 gene may be regulated by a single promoter [13].

Numerous studies have provided evidence suggesting that MiT/TFE transcription factors are important for the maintenance of cellular physiological and pathological processes. Among all the four members of the MiT/TFE family, TFEC is the least studied, and its function has not been widely investigated [14]. While MITF is critical for proliferation, survival and differentiation of melanocytes [3, 15]. And Mutations in MITF are also linked to the pigment and deafness disorder, Waardenburg syndrome type 2A [9]. And the presentation of TFE3 and TFEB is correlated with the development of osteoclasts [16], mast cell differentiation [17, 18], regulation of the expression of genes encoding critical metabolic regulators [19], activation of immune system [20] and control of allergic diseases [18, 21]. Additionally, TFEB is also essential for placental vascularization [22]. Furthermore, previous studies have revealed that the aberrant expression of several MiT/TFE family members is associated with different types of human cancers, such as renal carcinomas [23], alveolar sarcomas [24], and melanomas [25].

## Role of MiT/TFE family in lysosome and autophagy biogenesis

### Role of MiT/TFE in the transcriptional regulation of lysosome biogenesis

Lysosomes are crucial components of the cellular degradation and recycling system, and their correct function is required to maintain proper cell homeostasis [26]. These organelles are indeed involved in a number of essential cellular processes, including autophagy and lysosomal exocytosis [27]. Autophagosome clearance is initiated upon fusion with lysosomes and/or late endosomes, which introduce into the resulting autolysosome dozens of lysosomal acidic hydrolases and the acidification machinery necessary for enzyme activation and substrate digestion [28].

Lysosomes have long been considered as static organelles devoted to the terminal degradation of waste material; however, this concept has been challenged by subsequent discoveries that lysosomal biogenesis and function are subject to transcriptional regulation [1, 29]. The promoter analysis of lysosomal genes revealed that these molecules share a common 10-base E-box-like palindromic sequence (GTCACGTGAC), in most cases, localized within 200 base pairs of the transcription initiation site. This motif was named Coordinated Lysosomal Expression and Regulation (CLEAR) element [30]. TFE3 and TFEB directly bind to CLEAR elements on the promoters of several autophagic lysosomal genes to promote their expression [31, 32]. Accordingly, TFEB and TFE3 overexpression increases the number of lysosomes and levels of lysosomal enzymes, thus promoting lysosomal catabolic activity [2, 30], while the depletion of these genes abolishes the enhanced expression of lysosomal genes [2, 33]. Moreover, previous studies have also shown that TFEB can induce lysosomal exocytosis [34], a process by which lysosomes fuse to the plasma membrane and secrete their contents into the extracellular space, suggesting the transcriptional regulation of lysosome function which promotes intracellular clearance by TFEB. In a recent study, the role of MITF in the regulation of lysosomal biogenesis has been established in multiple cell types [35, 36]. MITF-A is localized at the lysosomal membrane, mimicking the transcriptional regulation of TFEB and TFE3 [37], and MITF-M drives the transcription of lysosomal markers and activates the CLEAR motif reporter in melanoma cells [36].

Notably, TFEB overexpression results in the enhanced degradation of bulk autophagy substrates, such as long-lived proteins [32], as well as the clearance of lipid droplets and damaged mitochondria [38, 39], suggesting that this transcription factor also plays a role in modulating organelle-specific autophagy, such as lipophagy and mitophagy. Previous studies have revealed that TFEB regulated peroxisome proliferator activated receptor gamma coactivator-1 alpha (PGC1α) expression, a regulator of mitochondrial biogenesis and oxidative stress, by directly binding to the PGC1α promoters in the liver [39] and cardiomyocyte [40]. While, absence of TFEB could affect mitochondrial complex II activity, increased oxidative stress, and decreased ATP production in skeletal muscle [41]. These results reveal an integrated view of how lysosomal signaling affects mitochondria. Interestingly, other recent studies suggested that the reciprocal positive feedforward TFEB-PGC1α signaling pathway plays a crucial role in the clearance of damaged mitochondria and mitochondrial biogenesis in skeletal muscle [42], and in mice with a Q311X Parikin mutation [43], as well as Huntington’s disease mouse model [44, 45]. Besides, the TFEB-dependent lysosomal alterations were also detected in others mitochondrial dysfunction, including the deletion of TFAM (loss of mtDNA), and PINK1 (mitophagy), and STUB1 (reduce mitochondrial protein degradation) and GCN5L1 (mitophagy), and inhibition of mitochondrial fission protein Drp1 (mitophagy), as well as mitochondrial complex I inhibition in various cell types and organs (T lymphocytes [46], neurons [38, 47, 48], heart [49], and mouse embryonic fibroblasts [50, 51]). All those experimental findings support that the biogenesis of mitochondria and lysosomes is regulated by the same MiT/TFE transcriptional factor. Similarly, TFE3 was also observed directly in regulation PGC1α expression in mitochondrial biogenesis. TFE3 ectopic expression induces PGC1α, while silencing TFE3 suppresses PGC1α expression in myotubes [52] and in liver [53]. In line with TFEB, MITF also regulates the expression of PGC1α in retinal pigment epithelium [54] and melanomas [55–57], further confirming the role of those transcription factors in energy metabolism (Table 1).

**Table 1:** Literature review of MiT/TFE family-mediated mitochondrial biogenesis and mitophagy

Core regulatory molecules or complex	Effect on mitochondrial	Related disease model	Reference
Mitochondrial transcription factor A (TFAM)	Controls mtDNA copy number	Lysosomal storage disorders, sphingolipidoses	[46]
Peroxisome proliferator coactivator-1 alpha (PGC1α)	Induces mitochondrial biogenesis, mitochondrial remodeling, respiration, gluconeogenesis and glucose transport, fatty acid oxidation, peroxisomal remodeling, and detoxification of reative oxygen species	Muscle wasting myopathies; Cardiac ischemia-reperfusion injury; Parkinson’s disease; Huntington’s disease; melanoma; obesity; Retinal pigment epithelium (RPE)-associated retinal degeneration; Non-alcoholic fatty liver disease (NAFLD)	[39, 40, 42–45, 47, 52–57]
Mitochondrial respiratory chain complex I	Initial and rate limiting enzyme in electron transfer chain	Parkinson’s disease (PD)	[50]
Mitochondrial respiratory chain complex II	Junction between oxidative phosphorylation and electron transport	Diabetes, obesity, and metabolic syndrome	[41]
Dynamin-related protein 1 (Drp1)	Key regulator of mitochondrial fission	Lethal dilated cardiomyopathy	[49]
PEN-induced putative kinase 1 (PINK1)	Recruits parkin resulting in ubiquitination of mitochondrial proteins	Parkinson’s disease (PD)	[38, 47]
GCN5-like Protein 1 (GCN5L1)	A putative nutrient-sensing regulator, controls mitochondrial removal by autophagy	Fatty liver, Type 2 diabetes	[51]
STIP1 homology and U-Box containing protein 1 (STUB1)	Promotes ubiquitin-mediated protein degradation	Neurodegenerative diseases	[48]

**Fig. 1:**
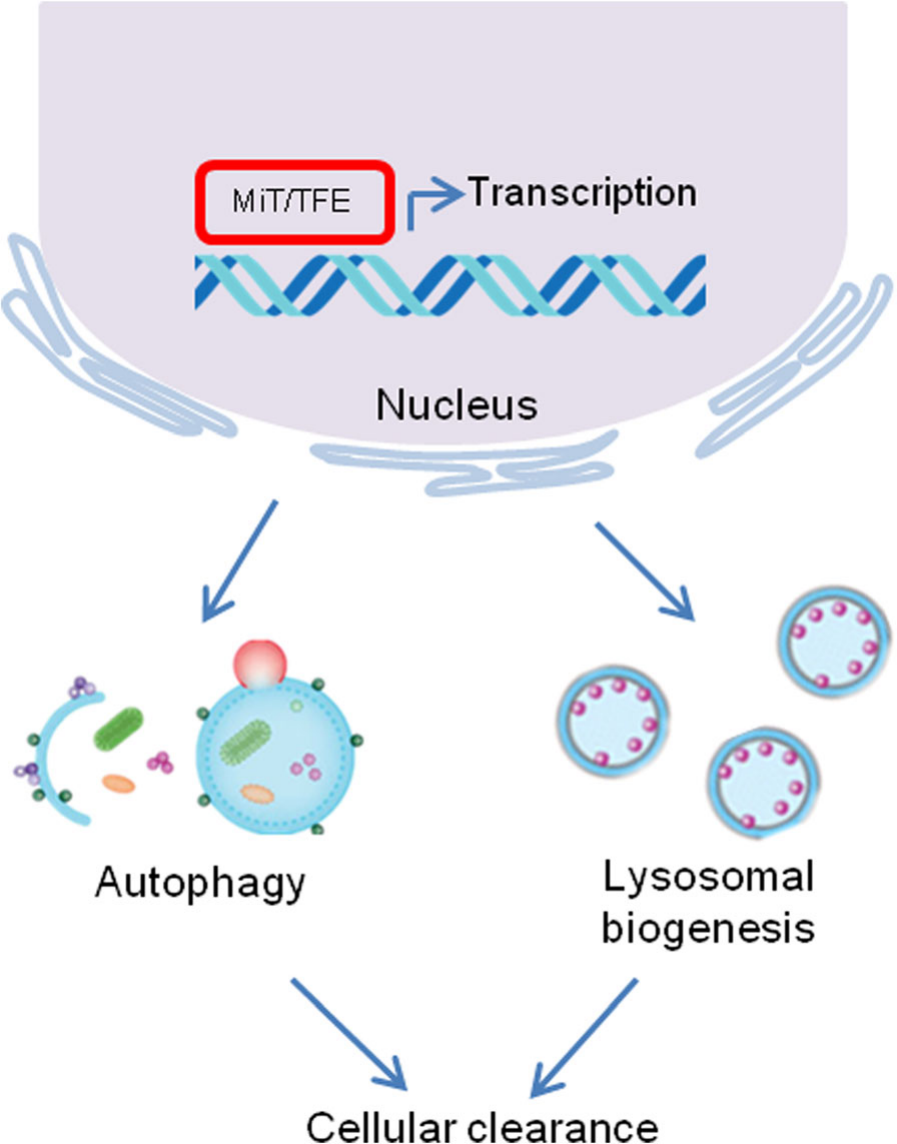
The MiT/TFE family of transcription factors regulates cellular clearance. The members of the MiT/TFE family participate in the regulation of cellular clearance via autophagy and lysosome biogenesis

These finding suggest that lysosomal biogenesis and function are coordinated by the transcriptional regulation of MiT/TFE family members (Fig. 1). And by modulating transcription factor activities, the cells can monitor lysosomal function and adapt to environmental signals.

### MiT/TFE family transcription factors participate in the regulation of autophagosome biogenesis

Autophagy is a critical catabolic pathway that is essential for the maintenance of cellular homeostasis via enabling the degradation of cellular components and recycling of important molecules under nutrient deprivation conditions [58, 59]. Autophagy is a complicated process that requires numerous autophagy-related genes (ATGs) to act at multiple stages of autophagy, including initiation, nucleation, and elongation steps [60]. Under normal conditions, the levels of ATG proteins are typically high, but some key autophagy regulators could be depleted under certain pathological conditions. For this reason, increasing the transcription of autophagy genes plays a pivotal role in cells under certain conditions, such as nutrient scarcity [9].

Subsequent studies have shown that TFEB is also a key transcriptional regulator of autophagy biogenesis, in addition to its crucial role in lysosomal function [32, 61]. In particular, TFEB directly binds to the promoter regions of numerous autophagy-related genes and promotes their expression [32]. These autophagy-related genes include LC3B, P62, VPS11, VPS18, UVRAG, WIPI and ATG9B. The overexpression of TFEB increases the number of autophagosomes in numerous cells, while the suppression of TFEB expression reduces autophagosome formation. [9]. At the same time, similar results are also obtained in the liver of GFP-LC3 transgenic mice, where activation of autophagy is observed following TFEB overexpression [32]. Therefore, by regulating lysosomal and autophagy biogenesis, TFEB coordinates a transcriptional program to control the main cellular degradative pathways and promote intracellular clearance. Importantly, TFEB does not regulate the basal transcription of its targets but rather enhances their transcriptional levels in response to environmental cues [1] and additional regulators of autophagic-lysosomal function have also been identified with the identification of TFEB [62].

Similar to TFEB, the overexpression of TFE3 in ARPE-19 cells promotes the transcriptional regulation of several critical autophagy-related genes, such as WIPI and ATG16L, suggesting a key role for TFE3 in the regulation of cellular processes [2]. Previous studies have shown that autophagy-related genes are not significantly correlated with MITF expression, while microarray analysis revealed that although many other autophagosomal genes were negatively associated with MITF, some autophagosomal genes were positively correlated with MITF, including UVRAG, VPS11, ATG3, SNCA, and AMBRA1 [32]. In GSEA analyses, many autophagy-related genes did not show a significant relationship with MITF, whereas lysosomal genes were highly correlated with this protein [36]. Furthermore, the overexpression of MITF-A, but not lysosomal genes, in RPE cells enhanced the activation of the autophagic process, adding an additional layer of mystery concerning the role of MITF in autophagy.

Taken together, these results suggested that MiT/TFE transcription factors play a pivotal role in the regulation of the autophagic process (Fig. 1), but the contribution of each member or certain MITF isoforms to autophagy biogenesis may depend on nutritional conditions, cell types and signaling inputs [14].

## Signaling involved in regulation of MiT/TFE family

### Regulation of MiT/TFE family member activities in mTORC1 signaling

The activity of TFEB is primarily modulated by post-translational modifications, protein-protein interactions and spatial organization [1]. Under normal conditions, TFEB is largely in an inactive state and remains in the cytoplasm [32, 63]. Under starvation or other extreme conditions, nuclear translocation of TFEB occurs rapidly with the transcriptional activation of its target genes.

The localization and activity of TFEB is mainly manipulated by phosphorylation. Two serine residues, that is, Ser142 [31] and Ser211 [31, 64], determine the subcellular localization of the TFEB protein. When both residues are phosphorylated, TFEB remains inactive in the cytosol, while variants of TFEB carrying Ser-to-Ala mutations of either Ser142 or Ser211 consistently show nuclear translocation and constitutive activation [64, 65]. Notably, the phosphorylation of Ser211 serves as a docking site for the chaperone 14–3-3 protein, which retains TFEB in the cytosol and prevents its nuclear translocation, likely by masking its nuclear localization signal [1, 65].

**Fig. 2:**
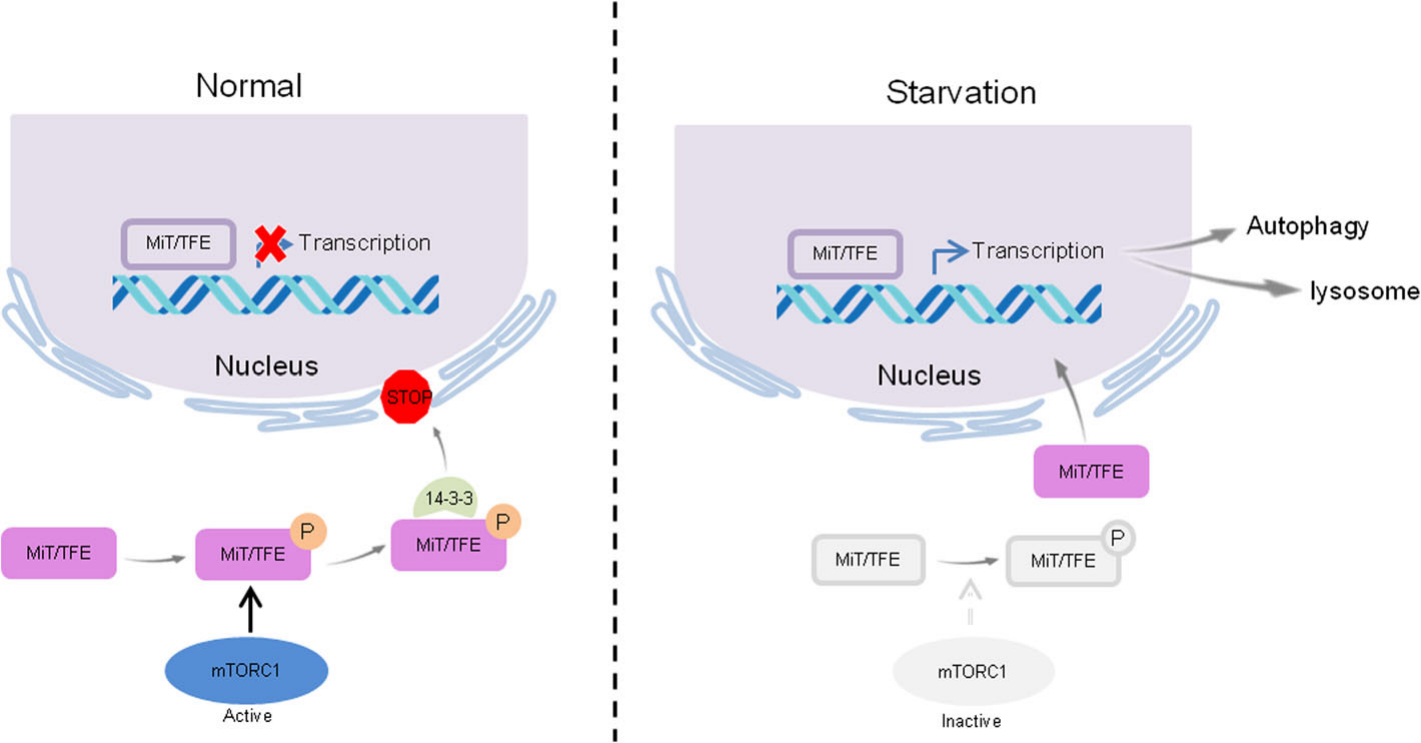
The mTORC1 signaling pathway is involved in the autophagy-lysosomal pathway via MiT/TFE transcriptional factors. Under normal nutrition conditions, the MiT/TFE members were phosphorylated by mTORC1, which were sequestered in the cytoplasm by 14–3-3 proteins. Upon starvation, mTORC1 was inactivated, leading to the dephosphorylation of MiT/TFE transcription factors, which resulted in the dissociation of the binding with 14–3-3 proteins that subsequently freely translocate to the nucleus where their transcriptional activation occurs in the autophagy-lysosome pathway [69]

Previous studies have demonstrated that the Mechanistic target of rapamycin complex 1 (mTORC1) is the main protein kinase regulating TFEB phosphorylation in most cell types [65, 66]. Remarkably, mTORC1 activation occurs at the lysosomal membrane. Under nutrition-full conditions, a mechanism involving the v-ATPase complex promotes the activation of the small Rag (Rag-related GTP-binding) GTPases, which recruit mTORC1 to the lysosomal membrane, thus promoting its activation through the small GTPase Rheb and inhibiting TFEB nuclear translocation [67, 68]. Intriguingly, active Rag GTPase can also bind TFEB and recruit it to the lysosomal membrane, thus enhancing TFEB phosphorylation by mTORC1 [37]. Upon starvation or lysosomal stress, mTORC1 is released from the lysosomal membrane and becomes inactive, and unphosphorylated TFEB accumulates in the nucleus, where it binds to CLEAR sequences and promotes subsequent gene expression [69] (Fig. 2). Interestingly, nutrient deprivation induces the release of lysosomal Ca^2+^ through Ca^2+^ channel mucolipin 1 (MCOLNI), thus activating the phosphatase calcineurin, which in turn dephosphorylates and promotes the nuclear translocation of TFEB [70]. The suppression of MCOLN1 expression inhibits lysosomal Ca^2+^ release and calcineurin activation, thus preventing TFEB activation and autophagy induction under nutrient insufficient conditions [70]. These results suggested that mTORC1 signaling was involved in the regulation of TFEB phosphorylation at the lysosomal membrane. However, it is unclear whether substrate phosphorylation by mTORC1 also occurs in lysosomes or whether active mTORC1 is released into the cytosol to exert its kinase activity [1].

A recent study also revealed that Ser122, another critical residue of TFEB, is important for TFEB subcellular localization by mTORC1 regulation [66]. These authors reported that Ser122 of TFEB was phosphorylated in an mTORC1-dependent manner. Specifically, TFEB nuclear localization following mTORC1 inhibition is blocked by Ser122 mutation of TFEB. And this mutation of TFEB also inhibits lysosomal biogenesis induced by Torin1, an inhibitor of mTORC1 [66]. These data reveal a novel mechanism of TFEB regulation by mTORC1, which is essential for lysosomal biogenesis.

Similar to TFEB, the activity of other MiT/TFE members can also be controlled by mTORC1 signaling. In the case of TFE3, its activity is also dictated by its nuclear localization, which is regulated by mTORC1 and nutrient levels. When nutrition is sufficient, the mTORC1 is recruited to the lysosomal membrane and become active, leading to phosphorylation of TFE3 at critical serine residues, such as Ser321, which cause TFE3 cytosolic retention; under nutrition-deprived conditions, TFE3 nuclear translocation occurs and activates the transcription of genes containing CLEAR promoter elements with mTORC1 signaling inhibition [2]. Additionally, a similar activation mechanism was also implicated in regulation of the localization of several MITF isoforms [37].

Taken together, these data demonstrated that the mTORC1 activation controls the activity and function of MiT/TFE transcription factors.

### Role of PKC in the regulation of MiT/TFE family members

**Fig. 3:**
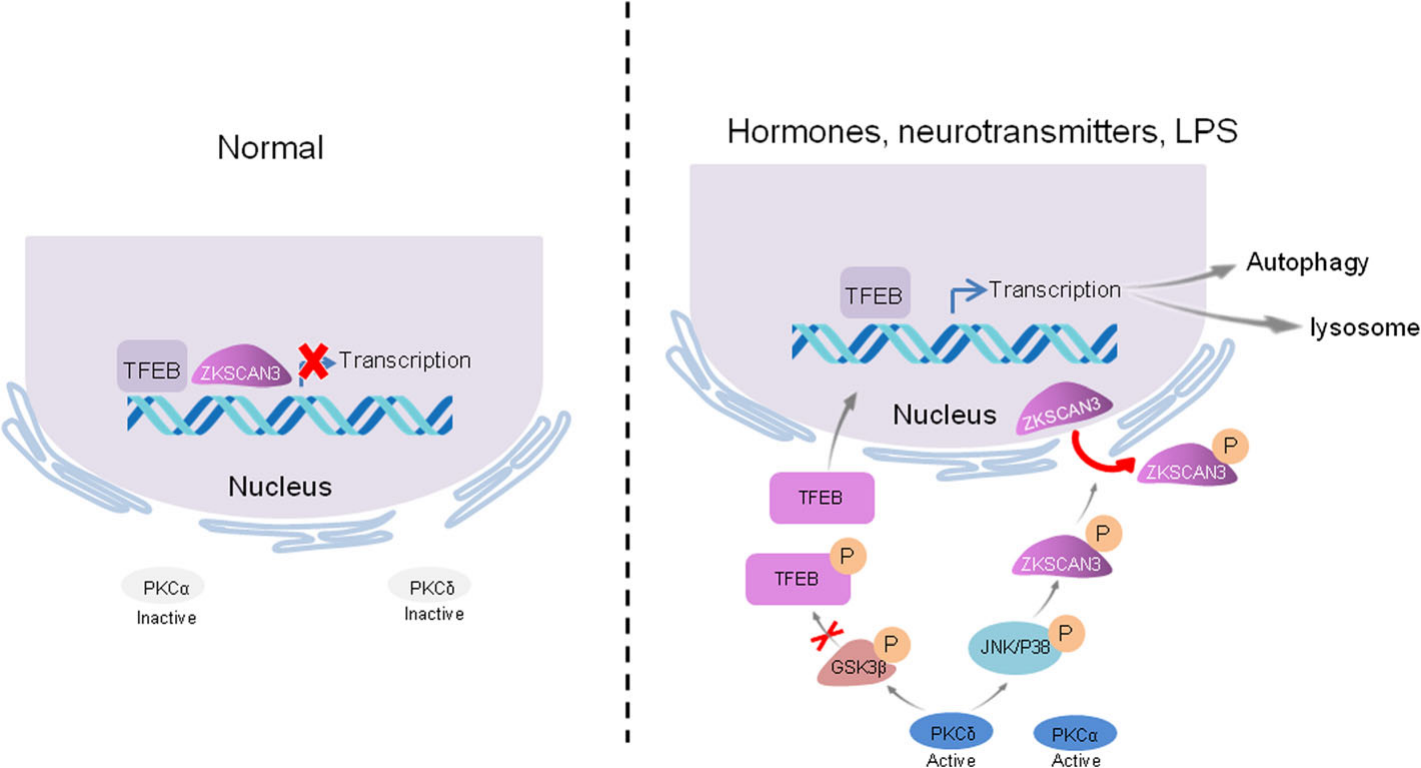
PKC triggered the transcriptional regulation of the TFEB-dependent autophagy-lysosome pathway. Under normal conditions, the PKC isoforms PKCα and PKCδ are inactivated. During hormone, neurotransmitter and bacterial lipopolysaccharide (LPS) stimulation, PKCα and PKCδ are activated, leading to their phosphorylation and inhibition of GSK3β, inhibiting the phosphorylation of TFEB serine sites needed for binding to cytoplasmic 14–3-3 proteins. TFEB nuclear translocation occurred and activated the expression of autophagy-lysosomal related genes. PKCδ activates JNK and P38, leading to the export of the repressor ZKSCAN3 from the nucleus to the cytoplasm,consequently alleviating transcriptional repression [74, 75]

Protein kinase C (PKC) signaling has been correlated with MITF in human melanogenesis [71, 72]. In osteoclasts, TFEB is directly phosphorylated at three serine residues located in its C-terminal region by PKCβ upon stimulation with receptor activator of nuclear factor κB ligand (RANKL) to promote lysosomal biogenesis and maintain its stability [73]. Additionally, recent evidence suggests that another PKC isoform can control lysosomal biogenesis without compromising mTORC1. These authors reported that PKC can couple the activation of the TFEB transcription factor, but not TFE3, with the inactivation of ZKSCAN3, a zinc-finger transcriptional factor, through two parallel signaling cascades. In the TFEB activation pathway, activated PKCδ phosphorylates and inactivates GSK3β, leading to the reduced phosphorylation of TFEB at Ser134 and Ser138 residues; this reduced phosphorylation is critical for the cytoplasmic sequestration of TFEB. Additionally, PKC further activates JNK and p38 MAPK, which in turn phosphorylate ZKSCAN3, leading to ZKSCAN3 translocation to the cytoplasm, which consequently alleviates the transcriptional repression of TFEB, with reduced the accumulation of polyQ aggregates and lipid droplets in HeLa cells. Moreover, injection of PKC inhibitor significantly increases Aβplaques in APP/PS1 mice, an animal model of Alzheimer’s disease, which further identify the evidence that PKC signaling is involved in regulation TFEB, thereby facilitating lysosomal clearance and mediating cellular adaptation to many extracellular cues [74, 75] (Fig. 3). Altogether, these data suggested that PKC is a master switch that controls two protein phosphorylation cascades to activate TFEB-mediated lysosomal gene expression without compromising mTORC1.

### AKT/PKB regulates the autophagy-lysosome pathway involved in MiT/TFE transcriptional factors

**Fig. 4:**
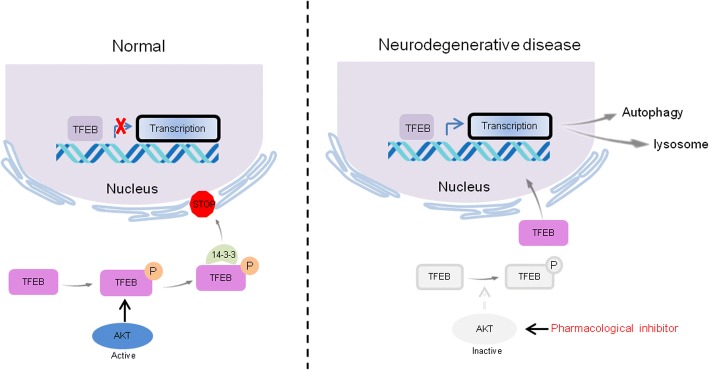
Signaling mechanism that regulates the TFEB nuclear translocation and activation involved AKT/PKB. Under normal conditions, AKT/PKB is activated, leading to phosphorylation, decreased nuclear translocation and TFEB activation. The inhibition of AKT activity with pharmacological inhibitors promotes TFEB nuclear translocation, which enhanced the transcriptional regulation of the autophagy-lysosome pathway and increased cellular clearance [81, 82]

Upon nutrition sufficiency, the nuclear translocation and activity of TFEB is inhibited by mTORC1 [64]. The mTORC1 is mainly controlled by several signal transduction pathways, such as Wnt and phosphatidylinositol 3(PI3K)-serine/threonine kinase AKT (also known as protein kinase B, PKB) signaling. The mTORC1 is activated downstream of AKT and PI3K kinases, and growth factor receptor signaling and inhibits autophagy under those growth promoting conditions [76, 77]. The canonical PI3K-AKT-mTORC1 pathway has been reported in several studies on TFEB and TFE3 activation [78–80]. However, mTORC1-independent TFEB activation via AKT inhibition, which promotes lysosome-mediated cellular clearance, has also been reported in recent years. Palmieri M et al. [81, 82] reported that TFEB activity is modulated by AKT phosphorylation at Ser467, and both AKT knockdown and the pharmacological inhibition of AKT promoted the nuclear translocation and stability of TFEB, as well as increased its ability to activate downstream target genes, thus promoting cellular clearance in a variety of models of genetic diseases, including patient-derived primary fibroblasts defective for PPT1, and TPP1M, and MFSD8, presenting with an impairment of lysosomal pathways (Fig. 4). In addition, a similar consequence was also observed for the TFEB paralogs, MITF and TFE3. Pharmacological inhibition of AKT promoted the nuclear translocation of these two transcriptional factors, suggesting the potential conservation of this regulatory pathway [81, 82]. Taken together, these findings provide novel perspectives for directly regulating cellular clearance by MiT/TFE transcriptional factors via AKT, without mTORC1 participation.

### The MiT/TFE family in cancer

The tight connection of TFEB and TFE3 with clear cell renal cell carcinoma (RCC) has been reported [83, 84]. MiT/TFE family translocation renal cell carcinoma (tRCC) comprises Xp11 tRCC and t(6:11)RCC, which were characterized by the rearrangement of the MiT transcription factors TFE3 and TFEB, respectively [85]. The fusion partner of TFEB in t(6:11) RCC is MALAT1 (also known as Alpha) [86, 87]. MALAT1 is a well-known long non-coding RNA (lncRNA) that fuses to TFEB upstream of the translation initiation codon ATG in exon 3, and this fusion promotes the translation of the encoded full-length TFEB protein [88]. In the case of TFE3, several RCC-associated fusion events have also been reported to occur between the TFE3 genes and various other genes, such as CLTC [89] and ASPL [90]. Intriguingly, the TFE3-ASPL fusion has also been reported in alveolar soft part sarcoma [90]. Moreover, multiple lines of evidence have shown that MITF plays an important role in melanoma biogenesis [15, 36]. Taken together, these results revealed that the upregulation of the MiT/TFE transcriptional network can drive tumorigenesis in many tissues.

Growing evidence has established the acceleration of autophagic and/or lysosomal dysfunction, with protein aggregates accumulation, as the major pathogenesis underlying these diseases [91, 92]. In recent years, researchers have paid more attention to whether the MiT/TFE family-mediated autophagic or lysosomal dysfunction also plays a critical role in cancers [93]. In human pancreatic ductal adenocarcinoma (PDA) cells, studies have revealed that autophagy induction occurs in PDA, relying on autophagy-lysosome function regulated by the MiT/TFE proteins, MITF, TFE3, and TFEB. Escape of those factors from inhibition by mTOR which control their cytoplasmic retention enables nuclear import increase, in turn, drives the expression of a coherent network of genes that facilitate the survival of PDA in response to nutrient stress [94]. Besides, to further extend this findings in primary patient-derived samples, Perera RM et al. also examined a series of early passage PDA cultures. Obviously, these cells have showed high level of autophagy, nuclear localization of MiT/TFE proteins as well as its mediated autophagy-lysosome gene expression. Furthermore, knockdown of TFE3 and MITF can abolish xenograft tumor growth of PDA cells, while MITF overexpression in *Kras* ^G12D^ mouse PanIN cells promoted tumorigenesis upon orthotopic injection [94]. Thus, those results suggested that MiT/TFE proteins are potent drivers of PDA pathogenesis with its requirement for increasing autophagy-lysosome function.

Similarly, up-regulation of MiT/TFE genes in cells and tissues from patients and mouse models of renal cell carcinoma, and melanoma enables cellular adaptation to nutrient availability and support the cellular metabolism of cancer cells, resulting in cell hyperproliferation and cancer growth [58]. Previous studies have revealed that mTORC1 can negatively regulate the activity of those MiT/TFE transcription factors, leading to their cytoplasmic retention. Here, Malta CD and his colleagues [58] reported that the MiT/TFE transcription factors can also in turn influence mTORC1 activity via RagD GTPase. They observed that hyperactivation and recruitment to lysosome of mTORC1 signaling and increased RagD levels in MiT/TFE overexpression cell and mouse model, whereas an opposite effect was also observed by MiT/TFE depletion, which was rescued by RagD overexpression. Notably, silencing of either MiT/TFE or RagD reduced cell proliferation in MiT/TFE overexpression tumor both in vivo and in vitro, and similar result was also obtained by mTORC1 inhibitor treatment. All those results suggested an MiT/TFE-RagD-mTORC1-MiT/TFE feedback circuit is critical for promoting tumor growth.

In parallel, the overexpression of TFEB was significantly correlated with poor prognosis in non-small cell lung cancer (NSCLC), Silencing of TFEB with siRNA could reduce the migration, but not proliferation, of NSCLC cells [95]. Furthermore, the enhancement of the autophagy flux by TFEB could rescue MITA (mediator of IRF3 activation)-induced cell death in breast cancer cells [96]. Taken together, these studies have demonstrated the MiT/TFE-controlled autophagy-lysosome function for the maintenance of intracellular homeostasis is critical for cancer.

## Conclusion

The identification of MiT/TFE transcriptional factors as master regulators of intracellular clearance and energy metabolism by orchestrating the genes involved in lysosomal-autophagic pathways has emphasized a mechanism by which the cell responds to environmental cues, such as nutrient deprivation. However, the mechanisms involved in the integration of multiple growth-stimulating and inhibitory signals to modulate an appropriate response and regulate a wide array of key cellular functions requires further investigation. Moreover, it remains unclear that how different signals can activate particular MiT/TFE members and promote responses to different environmental stimuli.

So far, we recognize that the TFEB and other MiT/TFE members, associated with autophagic or lysosomal dysfunction and the accumulation of toxic aggregates, present more and more close connection with cancers. This regulation mechanism enables cancer cells to maintain robust activation of anabolic pathways, thus promote cellular adaptation to stress, evidenced by the increase activation of MiT/TFE members in several cancers. However, the detail mechanism of MiT/TFE activation in cancer is still unclear and need further investigation. Therefore, understanding the specific functions of MiT/TFE transcriptional factors and the mechanism of their activation will certainly raise our cognizance of how cancer cell response so that they can survive under stress condition. And modulation of the activity of MiT/TFE transcriptional factors targeting aberrant autophagic and lysosomal functions may have a potential therapeutic benefit in cancer.

## Abbreviations

AKT: Serine/threonine kinase
ATG: Autophagy-related genes
CLEAR: Coordinated lysosomal expression and regulation
GSK3β: Glycogen synthase kinase 3 beta
HLH: Helix-loop-helix
IRF3: Interferon regulating factor 3
JNK: c-Jun N-terminal kinase
LDSs: Lysosomal storage disorders
MAPK: Mitogen activated kinase-like protein
MCOLNI: Ca^2+^ channel mucolipin 1
MiT/TFE: the Microphthalmia family of transcription factors
MITF: Melanogenesis associated transcription factor
mTORC1: the Mechanistic target of rapamycin complex 1
NSCLC: Non-small cell lung cancer
PDA: Pancreatic ductal adenocarcinoma
PKC: Protein kinase C
RANKL: Nuclear factor κB ligand
RPE: Retinal pigment epithelium
TFE3: Transcription factor E3
TFEB: Transcription factor EB
TFEC: Transcription factor EC
Zip: Leucine-zipper
ZKSCAN3: Zinc finger with KRAB and SCAN domains 3
